# Synthesis and Preclinical Evaluation of the Fibrin-Binding Cyclic Peptide ^18^F-iCREKA: Comparison with Its Contrasted Linear Peptide

**DOI:** 10.1155/2019/6315954

**Published:** 2019-06-27

**Authors:** Yin Zhang, Lijuan Wang, Sirui Yu, Kongzhen Hu, Shun Huang, Youcai Li, Hubing Wu, Hongsheng Li, Quanshi Wang

**Affiliations:** ^1^PET Center, Nanfang Hospital, Southern Medical University, Guangzhou, China; ^2^PET/CT Center, The First Affiliated Hospital of Guangzhou Medical University, Guangzhou, China

## Abstract

**Purpose:**

Cys-Arg-Glu-Lys-Ala (CREKA) is a pentapeptide which can target fibrin-fibronectin complexes. Our previous study has built a probe called iCREKA which was based on CREKA and has proved the feasibility and specificity of iCREKA by the fluorescence experiment. The purpose of this study is to achieve the ^18^F-labeled iCREKA and make preclinical evaluation of the ^18^F-iCREKA with comparison of its contrasted linear peptide (LP).

**Methods:**

CREKA, LP, and iCREKA were labeled by the Al^18^F labeling method, respectively. These ^18^F-labeled peptides were evaluated by the radiochemistry, binding affinity, in vitro stability, in vivo stability, micro-PET imaging, and biodistribution tests.

**Results:**

^18^F-NOTA-iCREKA was stable both in vitro and in vivo. However, ^18^F-NOTA-CREKA and ^18^F-NOTA-LP were both unstable. The FITC or ^18^F-labeled iCREKA could be abundantly discovered only in matrix metalloproteinases- (MMPs-) 2/9 highly expressed U87MG cells, while the FITC or ^18^F-labeled LP could also be abundantly discovered in MMP-2/9 lowly expressed Caov3 cells. Biodistribution and micropositron emission tomography (PET) imaging revealed that the U87MG xenografts showed a higher uptake of ^18^F-NOTA-iCREKA than ^18^F-NOTA-LP while the Caov3 xenografts showed very low uptake of both ^18^F-NOTA-iCREKA and ^18^F-NOTA-LP. The tumor-to-muscle (T/M) ratio of ^18^F-NOTA-iCREKA (9.93 ± 0.42) was obviously higher than ^18^F-NOTA-LP (2.69 ± 0.35) in U87MG xenografts.

**Conclusions:**

The novel CREKA-based probe ^18^F-NOTA-iCREKA could get a high uptake in U87MG cells and high T/M ratio in U87MG mice. It was more stable and specific than the ^18^F-NOTA-LP.

## 1. Introduction

The fibrin-fibronectin complexes have been reported as biomarkers for the early detection and diagnosis of cancer and micrometastasis [1]. CREKA, cysteine-arginine-glutamic acid-lysine-alanine, is a pentapeptide which can bind to fibrin-fibronectin complexes within various types of disease models including tumors [1, 2], atherosclerosis [3], and microthrombosis in myocardial ischemia-reperfusion [4, 5]. Although the CREKA can target the tumor tissues, it cannot enter into the tumor cells.

Cell-penetrating peptides (CPPs) are a family of various peptides that can pass through cell membranes and can be powerful transport vector tools for the intracellular delivery of a large variety of cargoes [6]. However, CPPs alone are not specific. Activatable cell-penetrating peptides (ACPPs) are CPPs which are inactive in normal environment but active in certain conditions [7]. ACPPs were more specific and easier to enter into cells than common CPPs [8]. Many reports have proved the feasibility of different kinds of ACPPs which were activated by matrix metalloproteinases (MMPs) [8, 9].

**Figure 1:**
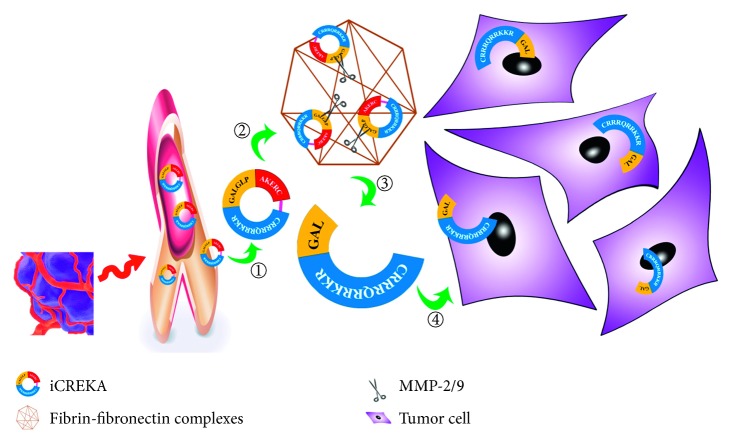
Schematic diagram of the iCREKA. ① The iCREKA reached at the tumor through the bloodstream based on the targeting ability of CREKA for fibrin-fibronectin complexes. ② The iCREKA was bound to the fibrin-fibronectin complexes. ③ While the cleavage sites PLGLAG remained intact, the iCREKA could not enter into tumor cells. In the MMP-2/9 highly expressed tumor, the cleavage sites PLGLAG were cut into two fragments by MMP-2/9. ④ One of the postcleavage fragments was composed of CCP (CRRRQRRKKR) and the residual cleavage sites GAL. This fragment could be endocytosed by the tumor cells.

Our previous study [10] has constructed a cyclic ACPP called iCREKA which consisted of the CREKA, the cell-penetrating peptides Tat_(49∼57)_ (RKKRRQRRR), and an enzyme cutting site PLGLAG (Figure 1(a)). The florescence assays revealed that it could enter into tumor cells by the enzyme digestion of MMP-2/9. However, the florescence imaging is not a common clinical imaging method. Some clinical imaging methods should be explored for the use of iCREKA.

PET can be used as a method for real-time visualization of pharmacological action on the molecular level. ^18^F-fluorine is an optimal radionuclide in clinical practice because of the moderate radioactive half-life and the high-quality images it provides on positron emission tomography/computed tomography (PET/CT) [11]. Fluorine-18-labeled iCREKA was preliminarily studied in our previous study. But the ^18^F-SFB method [12] in our previous study [10] or the ^18^F-NFP method to obtain fluorine-18-labeled peptides was proved to be time-consuming with low yield [13, 14]. It was reported that the Al^18^F labeling method was convenient and rapid, and the labeling yield of this method was relatively high [15]. Thus, we synthesized the fluorine-18-labeled peptides by the Al^18^F labeling method in this study.

The purpose of this study was to achieve the ^18^F-labeled iCREKA and to evaluate the ^18^F-labeled iCREKA in vitro and in vivo with comparison of its contrasted linear peptide (LP) which consisted of the same three components to iCREKA.

## 2. Materials and Methods

### 2.1. General

All chemicals obtained commercially were used without further purification. No-carrier-added ^18^F^−^ was obtained from GE PETtrace cyclotron (GE Healthcare, USA). Unmodified precursor peptides and FITC conjunct peptides were bought from ChinaPeptides Co., Ltd (China, Shanghai). NOTA conjunct peptides were bought from ChinesePeptides Co., Ltd (China, Hangzhou). MS spectrograms of the peptides were obtained from those two companies. The BALB/c nude mice (4–6 weeks old, 18–22 g) were purchased from the Experimental Animal Center of Southern Medical University. All animal studies were conducted in accordance with the protocols approved by the Experimental Animal Center of Southern Medical University.

Reverse-phase high-performance liquid chromatography (HPLC) analyses of the peptides were performed on a SHIMAZU system, including an SPD-M20A UV detector, a flow counts radiation detector (Bioscan), and a Shimadzu LC-10AD pump. HPLC solvents consisted of pure water containing 0.1% trifluoroacetic acid (solvent A) and acetonitrile containing 0.1% trifluoroacetic acid (solvent B). A ZORBAX Eclipse XDB-C18 column (5 *μ*m, 150 × 4.6 mm) was used with a flow rate of 1 mL/min. The HPLC gradient system began with an initial solvent composition of 95% solvent A and 5% solvent B for 2 min, followed by a linear gradient to 20% solvent A and 80% solvent B in 25 min, after which the column was reequilibrated.

### 2.2. Radiosynthesis of Fluorine-18-Labeled Peptides

#### 2.2.1. Manual Labeling

**Figure 2:**
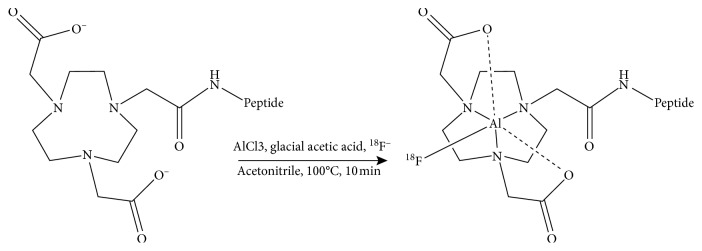
Schematic of the Al^18^F labeling method.

No-carrier-added aqueous ^18^F-fluoride was produced from the ^18^O^18^F reaction. 40 *μ*L deionized water, 6 *μ*L 0.01 mmol/L AlCl_3_, 5 *μ*L glacial acetic acid, 324 *μ*L acetonitrile, and 0.2 mg NOTA conjunct peptide were mixed well in a 1.5 mL EP tube by an ultrasonic oscillator. 555 MBq (15 mCi) ^18^F-fluoride (volume less than 100 *μ*L) was added into the EP tube. Then, the tube was sealed and placed in a thermostatic oscillator (Allsheng Instrument Co., Ltd, China). The reaction lasted for 10 min with a temperature of 100°C. The schematic of radiosynthesis was revealed in Figure 2. HPLC analysis of the mixture was then performed to assess the radiochemical yield. The mixture was then passed through a reverse-phase C-18 Sep-Pak plus extraction cartridge (Waters Corporation, USA) which was preconditioned with ethanol and water before use. The cartridge was washed by 10 mL of PBS and 20 mL of water. Then, 0.8 mL of ethanol containing 10 mM of hydrochloric acid was used to elute the product.

#### 2.2.2. Semiautomatic Labeling

This procedure was performed in a home-made multifunctional synthesis module (PET Co., Ltd., China) within a lead shielded hot cell. 100 *μ*l deionized water, 15 *μ*L 0.01 mmol/l AlCl_3_, 15 *μ*L glacial acetic acid, 780 *μ*L dimethyl formamide (DMF), and 0.5 mg NOTA conjunct peptide were mixed well and transferred into the reaction tube in the module. Then, ^18^F-fluoride (14.8–44.4 GBq/0.4–1.2 Ci) was transferred to the tube, and the radiosynthesis was proceeded with the temperature of 100°C for 10 min. The schematic diagram of the semiautomatic labeling was revealed in Supplementary Figure 1. The mixture was then transferred to a semipreparative HPLC to make separation.

### 2.3. Water Partition Coefficient

The water partition coefficient was reflected by the log*P* value of the fluorine-18-labeled peptides. 500 *μ*L of octanol and 500 *μ*L of pure water were added into an EP tube. 185 KBq (5 *μ*Ci) of fluorine-18-labeled peptides were added into the solution and well mixed. After centrifugating for 5 min at a speed of 1000 r/min, 300 *μ*L of supernatant liquid and 300 *μ*L of lower liquid were carefully pipetted into two *γ* countertubes, respectively. The logarithm of the radioactivity ratio of octanol to water was the log*P* value. The test was repeated four times for each fluorine-18-labeled peptide.

### 2.4. U87MG and Caov3 Nude Xenograft Models

The human primary glioblastoma cell line U87MG and the human ovarian adenocarcinoma cell line Caov3 were purchased from the Guangzhou Jennio Biotech Co., Ltd. These two cells were both grown in Dulbecco's Modified Eagle's Medium (DMEM) (ThermoFisher Scientific, USA) containing 10% fetal bovine serum (PAN biotech, Germany). Cells were passaged twice per week, at 80% confluence, and incubated in 5% CO_2_ at 37°C.

Animal procedures were performed according to the guideline of the Animal Ethics Committee of the Southern Medical University. Approximately 10 × 10^6^ U87MG cells and Caov3 cells were suspended in PBS and subcutaneously implanted in the right armpit of female BALB/c nude mice. Tumors were allowed to grow to a size of 0.5–1.5 cm in diameter.

*Pathological Verification*. The U87MG and Caov3 tumor tissues were pathologically analyzed by hematoxylin-eosin staining and immunohistochemical staining for assessing the expressions of MMP-2/9. The recombinant tissue inhibitor of metalloproteinases-1 (TIMP-1) (Abcam) was used as the primary antibody for immunohistochemical staining. A standard avidin-biotin-peroxidase technique was used for immunohistochemical staining.

### 2.5. Confocal Microscope Analysis

FITC-LP or FITC-iCREKA (0.2 mg) was sterilized by 2 mL DMEM medium and filtrated by an MF-Millipore™ Membrane Filter (Merck Millipore, USA). Subsequently, 1 mL of the solution was added to U87MG cells and Caov3 cells which adhered to glass bottom cell culture dishes or microscope cover glasses. After incubating at 37°C for 60 min, tumor cells were washed 5 times carefully with PBS and immobilized by 75% ethanol. The nucleuses were stained by 4′,6-diamidino-2-phenylindole (DAPI). The ﬂuorescence distribution in tumor cells was examined on a ZEISS LSM 880 (ZEISS, Germany) after final washing by PBS for 3 times. Fluorescence grayscale analyses were operated by ZEN 2 software (ZEISS, Germany) to make semiquantitative analyses.

### 2.6. Fluorine-18-Labeled Peptide Binding Affinity to Fibrin Clots

As previously reported [16], to synthesize fibrin clots, 75 *μ*L of fibrinogen solution (2 mg/mL) in 0.9% NaCl was added to four 1.5 mL EP tubes. 30 *μ*L of a 2.5 U/mL thrombin solution in 0.9% NaCl was subsequently added to these tubes. The tubes were placed in the thermostatic oscillator at 37°C for four hours, and the gels representative of fibrin clots were formed in the tubes. 370 KBq (10 *μ*Ci) fluorine-18-labeled peptide was diluted in 1 ml PBS. 100 *μ*L of the solution was added into each tube, respectively, and added into four empty tubes for reference. All tubes were incubated at 37°C for one hour. After that, the supernatant liquid was carefully pipetted. PBS was added and pipetted three times to remove any CREKA or peptides not bond to the fibrin clots. Finally, all tubes were measured by a CAPRAC®-R radioactive counter (Imaxeon) to obtain the bond-to-gross ratio.

### 2.7. In Vitro Stability

About 3.7 MBq (0.1 mCi) fluorine-18-labeled peptide was, respectively, added to a 1.5 mL EP tube which contained 1 ml phosphate buffer (PBS) and another tube which contained 1 ml mouse serum. These tubes were incubated at 37°C for 2 hours. Then, the PBS sample (50 *μ*L) and filtered serum sample were injected into the HPLC column to analyze the stability of the fluorine-18-labeled peptide.

### 2.8. In Vivo Stability

A nude with U87MG xenograft was injected with a dose of approximately 11.1 MBq (0.3 mCi) of ^18^F-labeled peptides via the tail vein. The mouse was euthanized at 30 min after injection, the tumor was carefully excised, and the blood was collected. The tumor was cut into pieces and made into homogenates by mixing 200 *μ*L normal saline. The mixture was centrifuged for 10 min at a speed of 12000 r/min. The supernatant liquid was carefully pipetted and transferred into an Amicon® Ultracentrifugal filter device for further centrifugation. The filtrate was injected into an HPLC column to analyze the in vivo stability of fluorine-18-labeled peptide.

### 2.9. Cellular Uptake Tests

U87MG cells and Caov3 cells were counted and uniformly inoculated into 24-well plates. The 24-well plates were marked, and cells were incubated for 24 hours in complete DMEM with 10% fetal bovine serum. Radio medium was prepared by adding 888 KBq (24 *μ*Ci) fluorine-18-labeled peptide into 15 mL DMEM without any serum. After incubation, the complete DMEM were poured and the radio medium were added in with 500 *μ*L/well. The remainder medium were collected into 4 *γ* countertubes with 500 *μ*L/tube to be representative of radioactivity gross. When the time arrived at 2, 5, 15, 30, 60, and 120 min, the radio medium were carefully pipetted out and the wells were carefully rinsed by 1 mL of PBS for 3 times. 4 wells were divided into a group corresponding to one time point. After the rinsing, SDS-NaOH solutions were added into the wells to dissociate the cells. The solutions were collected into *γ* countertubes to measure the radioactivity. The binding affinity was reflected by the radioactivity percentage, which was recorded as ID%, of the wells and the gross. The final results were recorded as the time-radioactivity curve including the six time points.

Fluorine-18 labeled peptides binding affinities after activation were also measured by this protocol while the radio-medium were mixed with activated MMP-2 (Abcam).

### 2.10. Small-Animal PET/CT

Small-animal PET/CTs were performed at the PET center of Southern Medical University Nanfang Hospital using a PET/CT scanner (Simens Inveon Micro-PET/CT). Tumor-bearing mice (*n* = 3/group) were intravenously injected with 5.55 MBq of ^18^F-NOTA-LP or ^18^F-NOTA-iCREKA under isoflurane anesthesia. A ten-minute static PET scan for each animal was acquired at 30, 60, and 120 min after the injection. 120 min dynamic scans were also obtained for ^18^F-NOTA-LP and ^18^F-NOTA-iCREKA (*n* = 2/group). The 3-dimensional ordered subset expectation maximization (3D-OSEM) algorithm was used for the PET reconstruction, and CT was applied for attenuation correction. Inveon Research Workplace (IRW) 3.0 software (Siemens, Germany) was used to measure the regions of interest (ROIs). The radioactivity concentrations were computed automatically, and the results were reflected by the unit of percent injected dose per gram (%ID/g). The maximum %ID/g of the ROIs was used for statistical analyses. Tumor-to-muscle (T/M) uptake ratios were calculated by dividing the radioactivity uptake in the tumor by the contralateral shoulder muscle.

### 2.11. Biodistribution

Three U87MG nude xenografts were injected with 1.85 MBq (0.05 mCi) ^18^F-NOTA-LP, and other three U87MG nude xenografts were injected with 1.85 MBq ^18^F-NOTA-iCREKA. These mice were euthanized at 1 h after injection. The tumor and organ samples were excised and transferred to preweighed *γ* counting tubes for weighing and gamma counting. The radioactivity associated with each sample tube was measured on the radioactive counter. Four empty tubes were measured as the background. Four tubes containing 37 KBq (1 *μ*Ci) ^18^F-NOTA-iCREKA in each tube were measured as the reference for conversion. The radioactivity counts (counts per minute) were corrected by the background and radioactive decaying. The %ID/g of each tissue and organ was calculated. The radioactivities in the stomach and intestines were including the contents in them.

### 2.12. Statistical Analysis

All results are expressed as mean ± SD. To determine the statistical significance of differences between the 2 groups, comparisons were made with the 2-tailed Student's *t* test for paired data. A *P* value of less than 0.05 was considered to be statistically significant.

## 3. Results

### 3.1. Chemistry

The mass spectrometry (MS) revealed that the molecular weights of our peptides were consistent with the theoretical molecular weight (Supplementary Figure 2). The retention time of the CREKA, LP, and iCREKA was 8.41, 12.20, and 11.58 min, respectively.

### 3.2. Radiochemistry

The radiochemical yields (decay-corrected) of the three peptides labeled by manual methods were 18.73∼33.41%, 13.28∼28.05%, and 8.78∼25.39% for ^18^F-NOTA-CREKA, ^18^F-NOTA-LP, and ^18^F-NOTA-iCREKA, respectively, after 10 min reaction time.

Most of the ^18^F-NOTA-CREKA could not be captured by the C-18 cartridge. ^18^F-NOTA-LP and ^18^F-NOTA-iCREKA could not be completely captured by the C-18 cartridge, and most of them could not be eluted from the cartridge. So the specific activity was calculated using the product of the semiautomatic labeling method. The specific activity of ^18^F-NOTA-CREKA was above 349.55 MBq/*μ*mol, and the specific activity of ^18^F-NOTA-LP and ^18^F-NOTA-iCREKA was above 466.33 MBq/*μ*mol and 179.53 MBq/*μ*mol, respectively.

The retention time of the ^18^F-NOTA-CREKA, ^18^F-NOTA-LP, and ^18^F-NOTA-iCREKA was 8.80 min, 11.78 min, and 11.92 min, respectively.

The log*P* values of ^18^F-NOTA-CREKA, ^18^F-NOTA-LP, and ^18^F-NOTA-iCREKA were −2.47 ± 0.05, −2.57 ± 0.02, and −2.51 ± 0.01.

### 3.3. In Vitro Stability

**Figure 3:**
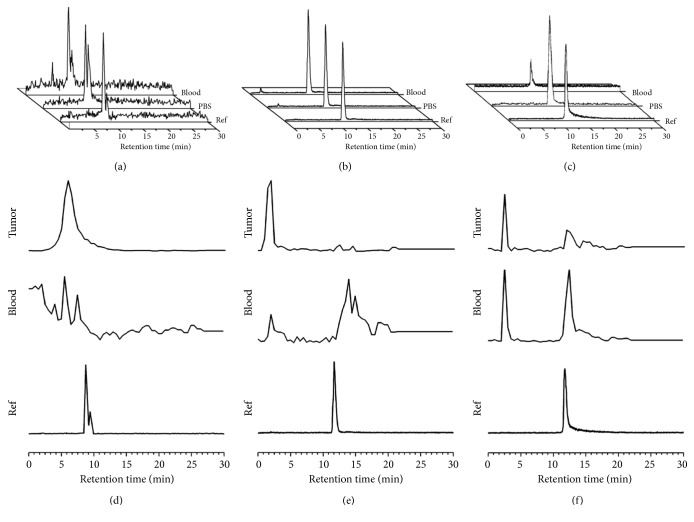
Retention time of the ^18^F-NOTA-CREKA (a), ^18^F-NOTA-LP (b), and ^18^F-NOTA-iCREKA (c) and their in vitro stability in PBS and serum. (d) ^18^F-NOTA-CREKA, (e) ^18^F-NOTA-LP, and (f) ^18^F-NOTA-iCREKA were the in vivo stability tests in the blood and tumor at 30 min after vein injection of fluorine-18 labeled peptides.

Defluorinations were discovered for the ^18^F-NOTA-CREKA and ^18^F-NOTA-LP, both in PBS and serum. The ^18^F-NOTA-iCREKA was consistently stable in PBS and serum during the 2-hour incubation. The HPLC analyses are also revealed in Figure 3.

### 3.4. In Vivo Stability

As revealed in Figures 3(d)–3(f), the three ^18^F-labeled peptides defluorinated in the blood and tumor. The ^18^F-NOTA-iCREKA kept 40.02% of the primary form stable in the blood and 24.29% in the tumor at 30 min after vein injection. There were 27.78% and 23.28% of the areas under the time-radioactivity curves representative of defluorination in the blood and tumor, respectively. By contrast, the ^18^F-NOTA-CREKA kept nearly none of the primary form stable in the blood and tumor. The ^18^F-NOTA-LP kept 0.94% of the primary form stable in the blood and nearly none in the tumor.

### 3.5. Fluorine-18-Labeled Peptide Binding Affinity to Fibrin Clots

The fibrin-binding percentages of ^18^F-NOTA-CREKA, ^18^F-NOTA-LP, and ^18^F-NOTA-iCREKA to fibrin clots were 69.31% ± 6.82, 67.09 ± 6.20, and 67.92 ± 6.71. There was no statistical difference among the three peptides (*P*=0.95).

### 3.6. Immunohistochemistry

**Figure 4:**
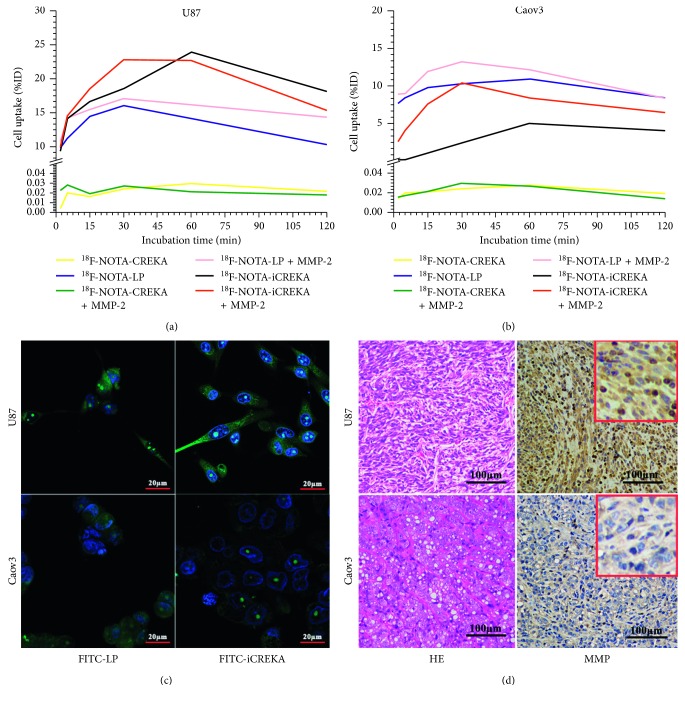
(a) Binding affinity of ^18^F-NOTA-CREKA, ^18^F-NOTA-LP, and ^18^F-NOTA-iCREKA to U87MG cells; (b) binding affinity of ^18^F-NOTA-CREKA, ^18^F-NOTA-LP, and ^18^F-NOTA-iCREKA to Caov3 cells; (c) confocal microscope analyses of FITC-LP and FITC-iCREKA in U87MG and Caov3 cells; (d) pathological verifications of the U87MG xenograft and Caov3 xenograft. The first column was H&E staining for U87MG xenograft and Caov3 xenograft, and the second column was immunohistochemical staining of MMP-2/9 expression for U87MG xenograft and Caov3 xenograft. The MMP-2/9 was highly expressed in U87MG tumor tissues (the brown portions) and was extremely lowly expressed in Caov3 tumor tissues.

The IHC results of MMP-2 were revealed in Figure 4(d). The U87MG tumor expressed MMP-2 abundantly, while the Caov3 tumor nearly expressed no MMP-2.

### 3.7. Confocal Microscope Analyses

As shown in Figure 4(c), the uptake of FITC-LP in U87MG and Caov3 cells were both high (92.67 ± 8.90 and 60.21 ± 4.36, respectively). By contrast, the uptake of FITC-iCREKA in U87MG cells was high (93.54 ± 8.43), while the uptake of FITC-iCREKA in Caov3 cells was relatively low (16.29 ± 1.67). In U87MG cells, there was no significant difference between the uptake of FITC-iCREKA and FITC-LP (*t* = 0.20, *P*=0.85). In Caov3 cells, the uptake of FITC-LP was obviously higher than the uptake of FITC-iCREKA (*t* = 27.24, *P* < 0.01).

### 3.8. Cellular Uptake Tests

As shown in Figure 4(a), the cellular uptake percentages of ^18^F-NOTA-iCREKA to U87MG cells were high and reached 23.90% as the peak at 60 min. As for the ^18^F-NOTA-LP to U87MG cells, by contrast, the cellular uptake percentages reached 16.05% as the peak at 30 min. For ^18^F-NOTA-CREKA, nearly no radioactivity was revealed in U87MG cells. After being activated by MMP-2, the cellular uptake of ^18^F-NOTA-LP and ^18^F-NOTA-iCREKA to U87MG cells were both increased, while still no radioactivity was revealed in U87MG cells for ^18^F-NOTA-CREKA.

For Caov3 cells, there was still nearly no radioactivity of ^18^F-NOTA-CREKA in tumor cells, no matter with or without the activation of MMP-2. The cellular uptake of ^18^F-NOTA-LP to Caov3 cells was high, and it became slightly higher by the activation of MMP-2. As a contrast, the cellular uptake of ^18^F-NOTA-iCREKA to Caov3 cells was low and became obviously increased by the activation of MMP-2 (Figure 4(b)).

### 3.9. In Vivo PET Imaging

**Figure 5:**
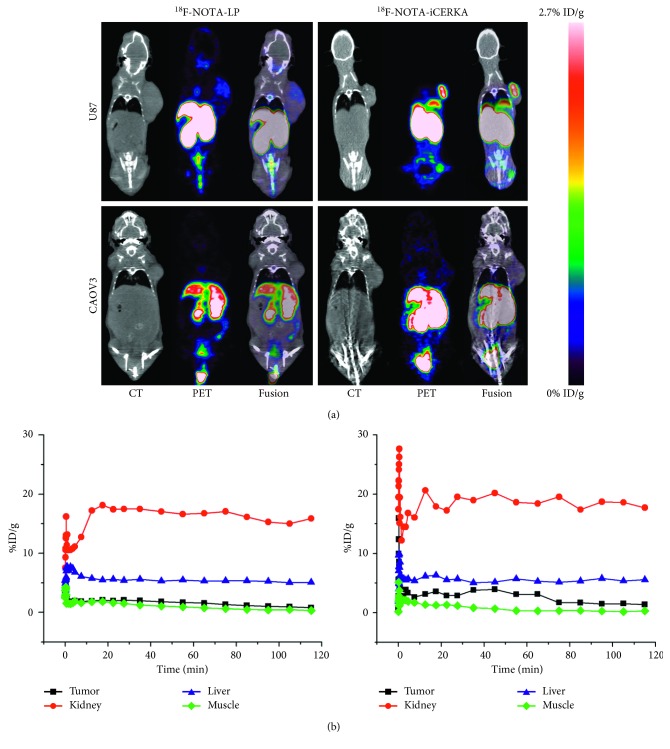
(a) Micro-PET imaging (60 min after tail vein injection) of ^18^F-NOTA-LP and ^18^F-NOTA-iCREKA in U87MG xenograft-bearing mice and Caov3 xenograft-bearing mice; (b) dynamic analyses of ^18^F-NOTA-LP and ^18^F-NOTA-iCREKA in U87MG xenograft-bearing mice.

In U87MG xenograft-bearing mice (Figure 5(a)), ^18^F-NOTA-LP and ^18^F-NOTA-iCREKA exhibited the highest uptake in the livers and kidneys. The U87MG tumor revealed moderate uptake of ^18^F-NOTA-iCREKA. By contrast, the uptake of ^18^F-NOTA-LP by the U87MG tumor was relatively low. At 60 min after radiotracer injection, the U87MG tumor revealed higher uptake of ^18^F-NOTA-iCREKA (2.9 ± 0.14) than ^18^F-NOTA-LP (1.45 ± 0.49) (*t* = 6.90, *P*=0.01). The T/M ratio for ^18^F-NOTA-iCREKA (6.48 ± 0.50) was higher than the T/M ratio for ^18^F-NOTA-LP (2.08 ± 0.50) (*t* = 15.28, *P* < 0.01).

In Caov3 xenograft-bearing mice (Figure 5(a)), ^18^F-NOTA-LP and ^18^F-NOTA-iCREKA exhibited a biodistribution similar to U87MG xenograft-bearing mice in most organs, with the highest uptake in the kidneys. The Caov3 tumor revealed nearly no uptake of radiotracer, no matter ^18^F-NOTA-LP (0.75 ± 0.40) or ^18^F-NOTA-iCREKA (0.68 ± 0.40). Radioactivities could be observed in the bone for two U87MG mice and two Caov3 xenograft-bearing mice.

Besides, the dynamic time-radioactivity curve could demonstrate the change of radioactivity in tumors and organs in real time (Figure 5(b)). At the 65 min, the T/M ratio of ^18^F-NOTA-iCREKA in the U87MG xenograft-bearing mice reached the maximum (9.93 ± 0.42). For ^18^F-NOTA-LP, the T/M ratio reached the maximum (2.69 ± 0.35) at 95 min.

### 3.10. Biodistribution

**Figure 6:**
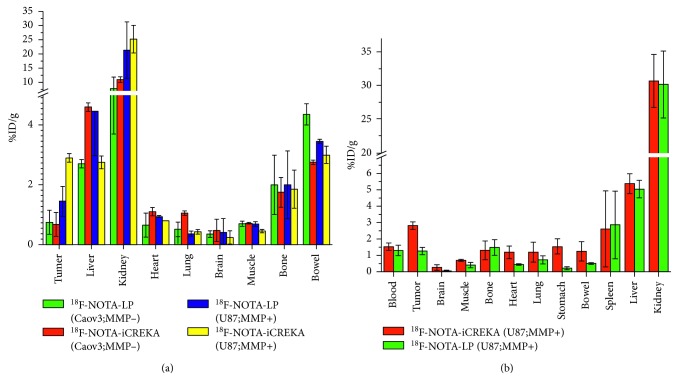
(a) %ID/g of tumors and organs of ^18^F-NOTA-LP and ^18^F-NOTA-iCREKA in U87MG xenograft-bearing mice and Caov3 xenograft-bearing mice on micro-PET; (b) %ID/g of tumors and organs in U87MG xenograft-bearing mice for ^18^F-NOTA-LP and ^18^F-NOTA-iCREKA in the biodistribution experiment.

The biodistribution (Figure 6(b)) showed similar results to micro-PET imaging (Figure 6(a)). The radioactivities of ^18^F-NOTA-iCREKA were higher than the radioactivities of ^18^F-NOTA-LP in U87MG xenografts (*t* = 8.47, *P* < 0.01). T/M ratio for ^18^F-NOTA-LP was 3.01 ± 0.37, while the T/M ratio for ^18^F-NOTA-iCREKA was 4.31 ± 0.22, and there were statistic differences between them (*t* = 6.71, *P*=0.04). The %ID/g of the spleen, which was difficult to be delineated on micro-PET imaging, was relatively high (2.74 ± 1.97).

## 4. Discussion

CREKA-based molecular probes can provide the ability of targeting fibrin-fibnectin complexes in tumors [1, 2], atherosclerosis [3], and microthrombosis [4, 5]. In this study, we developed the first CREKA-based PET probe ^18^F-NOTA-iCREKA for tumor imaging and compared it with its linear form ^18^F-NOTA-LP and ^18^F-NOTA-CREKA.

^18^F-NOTA-iCREKA revealed superior stability than ^18^F-NOTA-CREKA and ^18^F-NOTA-LP, both in vivo and in vitro. ^18^F-NOTA-CREKA and ^18^F-NOTA-LP revealed slightly defluorination in PBS and serum, while ^18^F-NOTA-iCREKA kept stable all the way. The in vivo stability tests proved the superior stability of ^18^F-NOTA-iCREKA than ^18^F-NOTA-CREKA and ^18^F-NOTA-LP again. All these probes were defluorinated in vivo. There was no ^18^F-NOTA-CREKA left in blood and tumor at 30 min after injection, and the ^18^F-NOTA-LP kept in blood and tumor was both less than 1%. By contrast, there was nearly no decomposition in blood and partial decomposition in tumor for ^18^F-NOTA-iCREKA. So the ^18^F-NOTA-iCREKA could keep stable before it reached the tumor and then took effect after digestion by MMP-2/9 in the tumor stroma. These were in accordance with the previous studies that reported the superior stability of the cyclic peptide over the linear peptide. The cyclic structure may protect the weak C-H bonds from being attacked [17, 18].

The in vitro binding affinity to fibrin clots revealed that the three peptides owned similar ability to bind to fibrin clots. The fibrin-targeting ability was not obviously influenced by the structural changes. Furthermore, cellular uptake tests revealed that the uptake levels of ^18^F-NOTA-LP and ^18^F-NOTA-iCREKA by U87MG cells, which was highly expressed MMP-2/9, were both high (the maximums of %ID were 23.90% and 16.05%, respectively), while the uptake levels of ^18^F-NOTA-CREKA were extremely low (the maximums of %ID was 0.03%). For the MMP-2/9 lowly expressed Caov3 cells, the uptake level of ^18^F-NOTA-LP was high (10.90%), while the uptake level of ^18^F-NOTA-iCREKA was relatively low (5.02%). Still, the uptake levels of ^18^F-NOTA-CREKA were extremely low (0.03%). The uptake levels of ^18^F-NOTA-LP and ^18^F-NOTA-iCREKA could both be enhanced by MMP-2. These tests revealed the superior cellular uptake of ^18^F-NOTA-iCREKA than most reported PET probes and proved the superior specificity of ^18^F-NOTA-iCREKA than ^18^F-NOTA-LP. The CREKA could bind to fibrin-fibronectin complexes in the tumor stroma but had no ability to enter into cells and thus was washing out when rinsing with PBS. A lot of ^18^F-NOTA-LP being found in Caov3 cells may be due to the unrestricted activity of CPP in the state of the linear structure. While the CPP was changed into a different conformation, the cell-penetrating activity was limited [19, 20]. The ^18^F-NOTA-iCREKA not only kept the binding affinity to fibrin-fibronectin complexes but also restricted the transmembrane ability before activation by MMP-2. This could effectively prevent the probes from entering into normal cells.

Since the ^18^F-NOTA-CREKA was not stable and could not enter into tumor cells, we did not further evaluate the ^18^F-NOTA-CREKA by micro-PET and biodistribution. Although the %ID/g of the U87MG tumor was moderate for ^18^F-NOTA-iCREKA on micro-PET imaging and in biodistribution, the T/M ratio was high. By contrast, the %ID/g of the U87MG tumor and T/M ratio for ^18^F-NOTA-LP on micro-PET imaging and in biodistribution was both relatively low. These proved the superiority of ^18^F-NOTA-iCREKA over the ^18^F-NOTA-LP for PET imaging. Biodistribution and micro-PET imaging revealed that uptake in the bone was heterogeneous and relatively high, indicating a different level of defluorination for the ^18^F-NOTA-LP and ^18^F-NOTA-iCREKA. These were corresponding to our in vivo stability tests but conflicting to the reported studies, which revealed that the Al^18^F conjunct peptides were difficult to be defluorinated [15, 21]. Soluble proteases such as MMP have the potential disadvantage of gradually leaking from the tumor into the general circulation, where they would contribute to the background signal and reduce contrast [22]. The inconstant results in biodistribution may be caused by the numerous influence factors, such as the errors in weighing the tumor and organs and the errors in measuring the radioactivity. Even a little error in measurement could cause a big difference in result. So the maximum %ID/g of the ROIs on micro-PET imaging seemed to be more reliable.

While compared with other commonly used radiotracers labeled by the Al^18^F method, our ^18^F-NOTA-iCREKA revealed a lower tumor uptake but a higher tumor-to-muscle ratio than the ^18^F-NOTA-PRGD_2_ (2.9 ± 0.1%ID/g vs. 5.3 ± 1.7%ID/g and about 9.9 vs. 2.9 at 60 min after injection [23]). The higher tumor-to-muscle ratio could lead to better image quality. Besides, although the log*P* value of ^18^F-NOTA-iCREKA was −2.51 ± 0.01, excretions of ^18^F-NOTA-iCREKA via the liver and kidney were both more than the corresponding results for ^18^F-NOTA-PRGD_2_ (about 4 vs. 3%ID/g and about 27 vs. 6%ID/g). This may be due to the differences in hydrophilia between ^18^F-NOTA-iCREKA and ^18^F-NOTA-PRGD_2_. Improving the hydrophilia could speed up the excretion and reduce the biotoxicity.

Many studies had proved that the CREKA could target fibrin-fibronectin complexes and provide a noninvasive way for in vivo imaging of tumor stroma [1, 4, 5, 24]. These studies reported that CREKA-conjugated nanoparticles, liposome, and micelles were stably feasible in vitro and in vivo. However, in our study, the in vivo stability of ^18^F-NOTA-CREKA was bad. Our cyclic ACPP based on CREKA could not only bring the ability of endocytosis but also improve the stability while keeping the fibrin-fibronectin complexes targeting ability. The ability of endocytosis could potentially provide the ability of taking therapeutic drugs into tumor cells. Besides the application in tumor diagnosis, our cyclic ACPP based on CREKA could also be used in molecular imaging of thrombosis, like other fibrin-binding probes [25–28].

It is reported that the radiochemical yield of the Al^18^F labeling method is relatively low but the in vivo stability of the Al^18^F-labeled radiotracer is high [15, 21, 29]. Radiochemical yields of our three peptides were relatively low, which were consistent with these studies. Adjusting the reaction system to the optimal dosage may improve the radiochemical yield.

## 5. Conclusion

In this study, we successfully synthesized a novel fluorine-18-labeled cyclic ACPPs based on CREKA, which was called iCREKA, and further evaluated it with a comparison of its linear status peptide (LP). ^18^F-NOTA-iCREKA was stable in vitro and could get high uptake in MMP-2/9 highly expressed U87MG tumor cells while the uptake in MMP-2/9 lowly expressed Caov3 cells was extremely low. Micro-PET imaging and biodistribution of ^18^F-NOTA-iCREKA showed moderate uptake and high T/M ratio in the U87MG tumor. The ^18^F-NOTA-iCREKA was more stable and could get higher uptake and higher T/M ratio than the ^18^F-NOTA-LP. This fibrin-binding iCREKA could be a potential probe and carrier of therapeutic drugs for the MMP-2/9 highly expressed tumor.

## Data Availability

The data used to support the findings of this study are included within the article and the supplementary information file(s).

## Supplementary Materials

Supplementary Materials The concise descriptions of Supplementary Materials were listed as below. Supplementary Figure 1: schematic diagram of the semiautomatic labeling Supplementary Figure 2: mass spectrometry (MS) analyses of our peptides. Sequences of these peptides are as follows: LP: CREKAPLGLAGRKKRRQRRRCK; iCREKA: CREKAPLGLAGRKKRRQRRRCK (remark : Disulfide bridges C1-C21); FITC-CREKA: FITC-(Acp)-CREKA; FITC-LP: CREKAPLGLAGRKKRRQRRRCK (FITC); FITC-iCREKA: CREKAPLGLAGRKKRRQRRRCK (FITC) (remark : Disulfide bond); NOTA-CREKA: NOTA-CREKA; NOTA-LP: CREKAPLGLAGRKKRRQRRRCK (NOTA); NOTA-iCREKA: CREKAPLGLAGRKKRRQRRRCK (NOTA) (Cys&Cys bridge).
