# Supplementary material for: Synthesis and Preclinical Evaluation of the Fibrin-Binding Cyclic Peptide 18F-iCREKA: Comparison with Its Contrasted Linear Peptide
Source: Contrast Media Mol Imaging. 2019 Jun 27;2019:6315954. doi: 10.1155/2019/6315954 (PMC6620859; doi:10.1155/2019/6315954)
Supplement: Supplementary Materials — The concise descriptions of Supplementary Materials were listed as below. Supplementary Figure 1: schematic diagram of the semiautomatic labeling Supplementary Figure 2: mass spectrometry (MS) analyses of our peptides. Sequences of these peptides are as follows: LP: CREKAPLGLAGRKKRRQRRRCK; iCREKA: CREKAPLGLAGRKKRRQRRRCK (remark : Disulfide bridges C1-C21); FITC-CREKA: FITC-(Acp)-CREKA; FITC-LP: CREKAPLGLAGRKKRRQRRRCK (FITC); FITC-iCREKA: CREKAPLGLAGRKKRRQRRRCK (FITC) (remark : Disulfide bond); NOTA-CREKA: NOTA-CREKA; NOTA-LP: CREKAPLGLAGRKKRRQRRRCK (NOTA); NOTA-iCREKA: CREKAPLGLAGRKKRRQRRRCK (NOTA) (Cys&Cys bridge). [file 6315954.f1.docx]

Supplemental materials


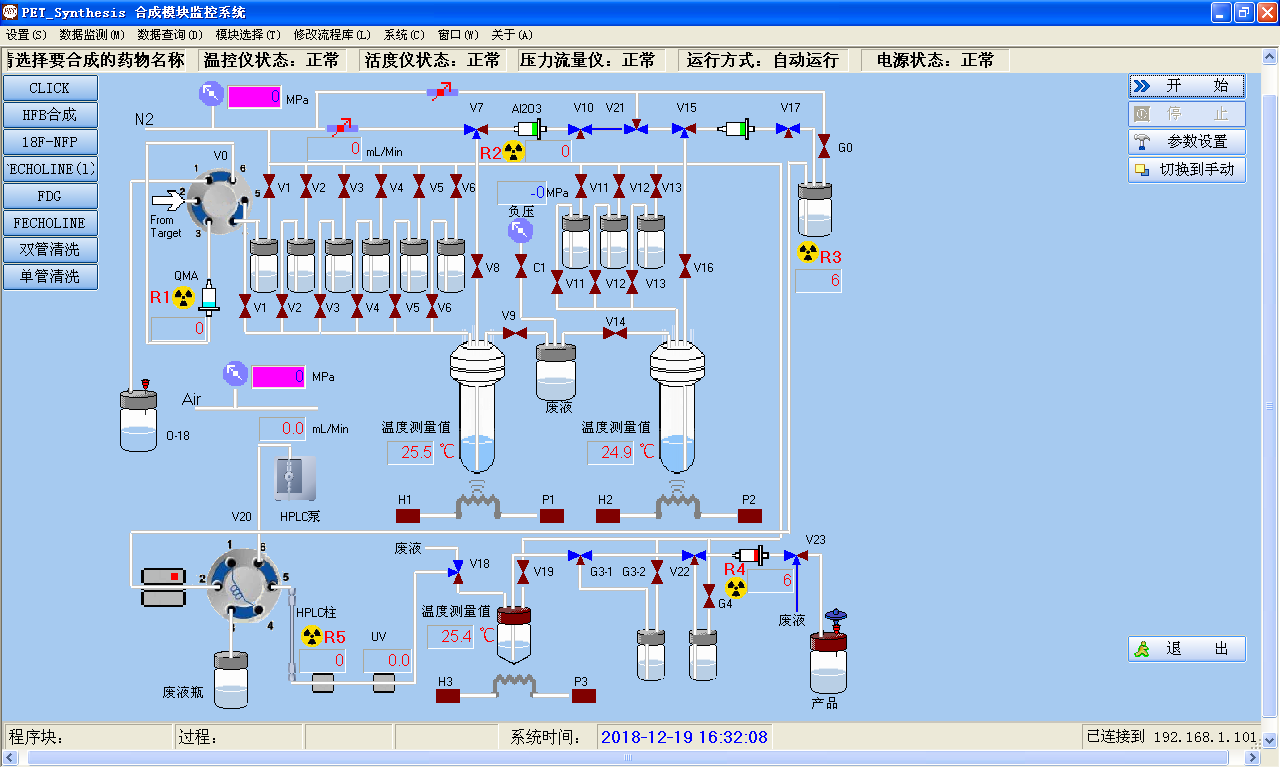


Supplemental figure 1: The schematic diagram of the semi-automatic labeling


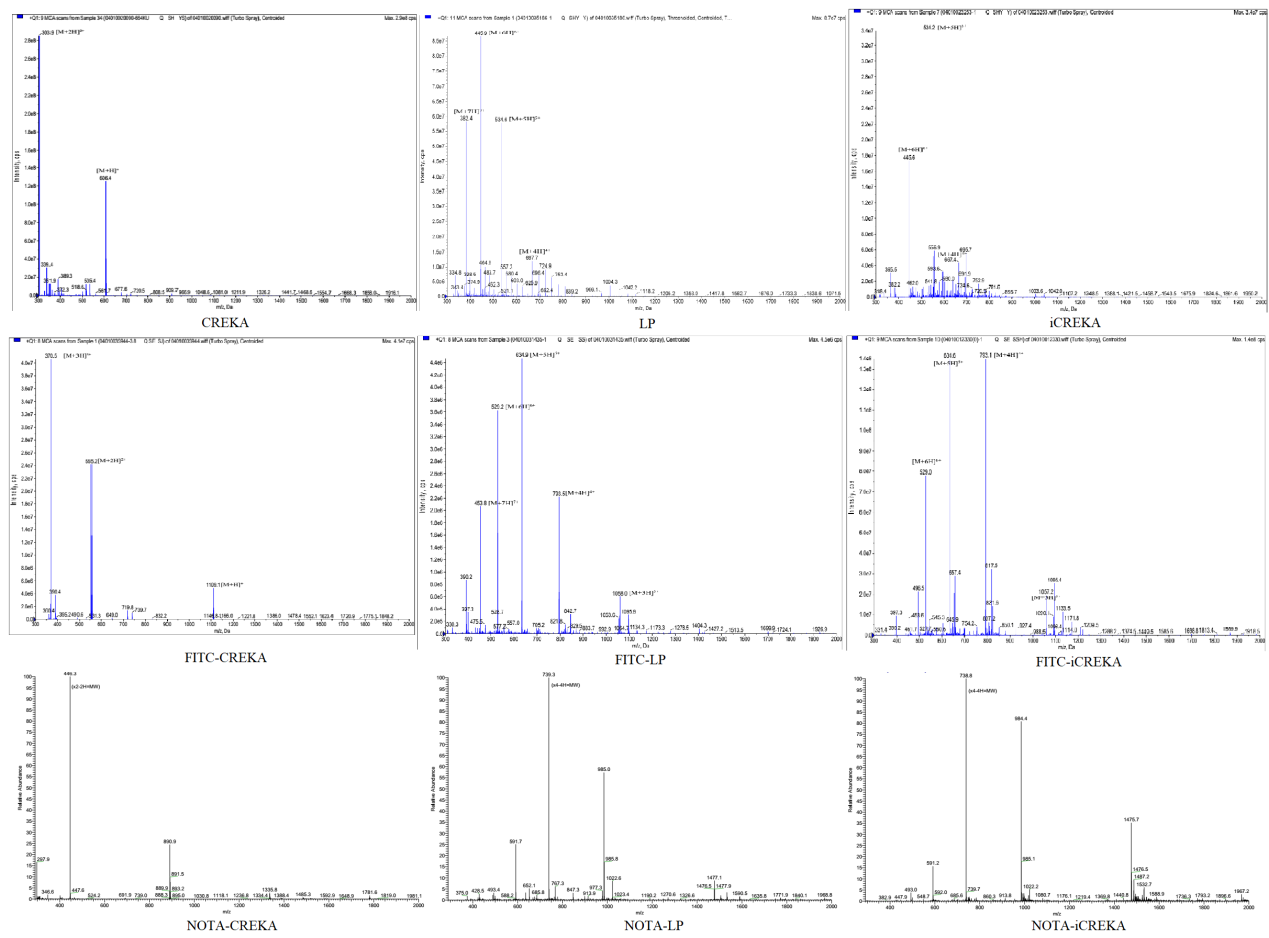


Supplemental figure 2: The mass spectrometry (MS) analyses of our peptides.

Sequences of these peptides are:

LP: CREKAPLGLAGRKKRRQRRRCK

iCREKA: CREKAPLGLAGRKKRRQRRRCK (Remark:Disulfide bridges C1-C21)

FITC-CREKA: FITC-(Acp)-CREKA

FITC-LP: CREKAPLGLAGRKKRRQRRRCK(FITC)

FITC-iCREKA: CREKAPLGLAGRKKRRQRRRCK(FITC) (Remark:Disulfide bond)
